# Uterine Myxoid Tumor: A Case Report on a Mysterious and Unexpected Diagnosis

**DOI:** 10.7759/cureus.33568

**Published:** 2023-01-09

**Authors:** Reham A Khubrani, Abdulmohsen Alkushi, Mona M Mahjoub

**Affiliations:** 1 Department of Basic Sciences, Princess Nourah Bint Abdulrahman University, Riyadh, SAU; 2 Department of Pathology and Laboratory Medicine, King Abdullah bin Abdulaziz University Hospital, Riyadh, SAU; 3 Department of Pathology and Laboratory Medicine, King Abdulaziz Medical City Riyadh, Riyadh, SAU; 4 College of Medicine, King Saud Bin Abdulaziz University for Health Sciences, Riyadh, SAU; 5 Department of General Obstetrics and Gynecology, King Abdullah bin Abdulaziz University Hospital, Riyadh, SAU

**Keywords:** uterine myxoid neoplasm, uterine malignancy, uterine fibroid, imt, inflammatory myofibroblastic tumor (imt)

## Abstract

Inflammatory myofibroblastic tumors (IMTs) are soft-tissue tumors that are rarely malignant. They can occur throughout the body but are rarely seen in the female genital tract. To our knowledge, the literature reports only a few cases of uterine IMTs. This article describes a 60-year-old female with a uterine IMT presumed to be a fibroid. The correct diagnosis was made based on a combination of gross appearance, microscopic findings from multiple hematoxylin and eosin-stained tumor slides, and immunohistochemical staining. We also provide a literature review and compare our findings to other reported cases.

## Introduction

An inflammatory myofibroblastic tumor (IMT) is a well-known, soft-tissue tumor classified by the World Health Organization as an “intermediate (rarely metastasizing) fibroblastic myofibroblastic tumor” [1]. It can occur in nearly any anatomic location, including most organs, though it is rare in the female genital tract, and it has nonspecific symptoms [2]. To our knowledge, only a few cases of uterine IMTs are reported in the literature. This article describes a case of uterine IMT that was originally presumed to be a fibroid.

## Case presentation

**Figure 1:**
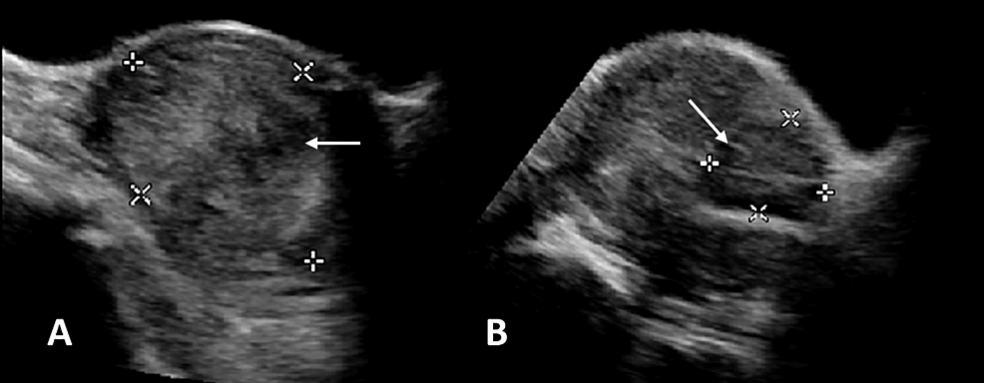
Ultrasound images of the uterine intramural (A) and subserosal (B) masses show well-defined boundaries and similar echogenicity.

A 60-year-old female with a history of three vaginal deliveries and menopause for seven years presented with intermittent vaginal bleeding and abdominal pain for over a month. Transabdominal pelvic ultrasonography imaging showed an intramural 6.5-cm hypoechoic mass and two subserosal hypoechoic masses measuring 3.0 cm and 4.0 cm. The endometrial thickness was 3 mm. The radiologist diagnosed the three masses as uterine fibroids, and no further imaging was performed (Figure 1).

The patient underwent an endometrial biopsy, which revealed benign proliferative endometrium. Due to longstanding vaginal bleeding leading to anemia, she underwent a total hysterectomy with bilateral salpingo-oophorectomy under spinal anesthesia. The procedure was performed via a Pfannenstiel skin incision with the patient in a supine position. The specimen was resected smoothly without complications and sent to the histopathology lab in formalin. The surgery was ended after confirming the absence of any seedings or local spread.

**Figure 2:**
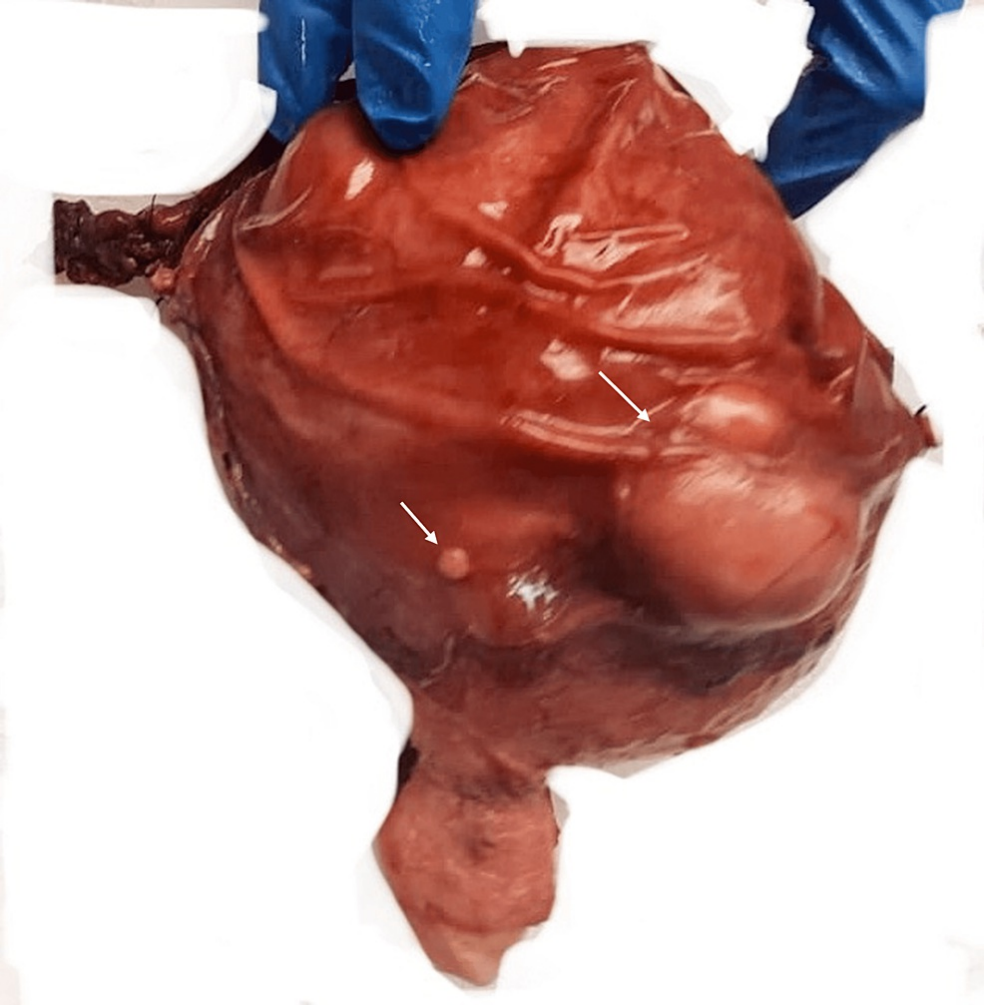
Gross image showing the uterine serosal surface with subserosal bulgings.

**Figure 3:**
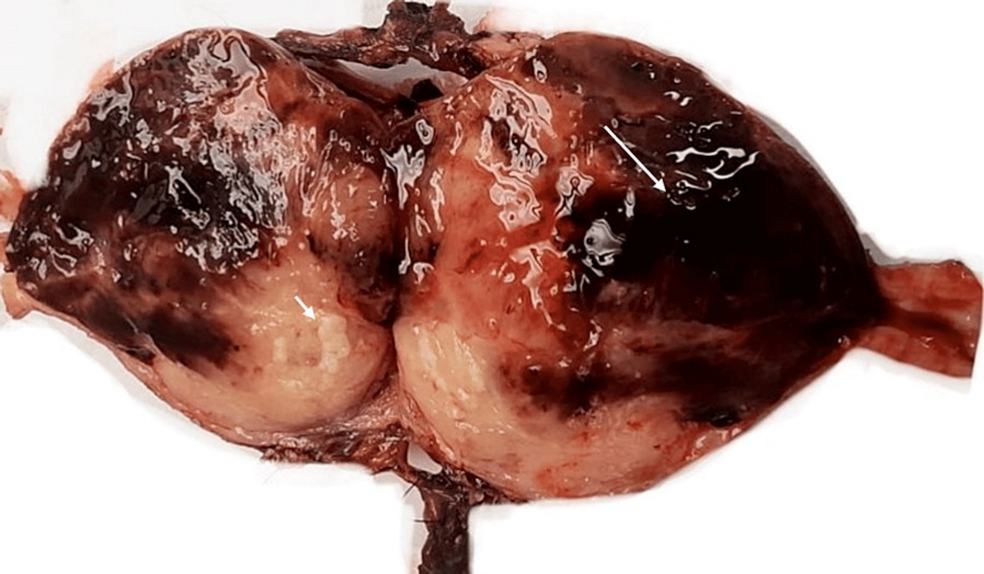
Gross image showing necrotic (short arrow) and hemorrhagic (long arrow) cut surface of tumor in the uterine cavity.

Our lab analyzed the total hysterectomy with a bilateral salpingo-oophorectomy specimen consisting of an intact uterus with smooth, shiny serosa and several subserosal protuberances ranging from 0.4 to 4.0 cm. Upon opening the uterus, we found a large, heterogeneous mass occupying the entire uterine cavity except for the cervix. The mass was soft and focally infiltrative with a myxoid, hemorrhagic, and necrotic cut surface. The subserosal masses had a homogeneous, white, firm, solid-cut surface (Figures 2, 3). The cervix, ovaries, and fallopian tubes were unremarkable.

**Figure 4:**
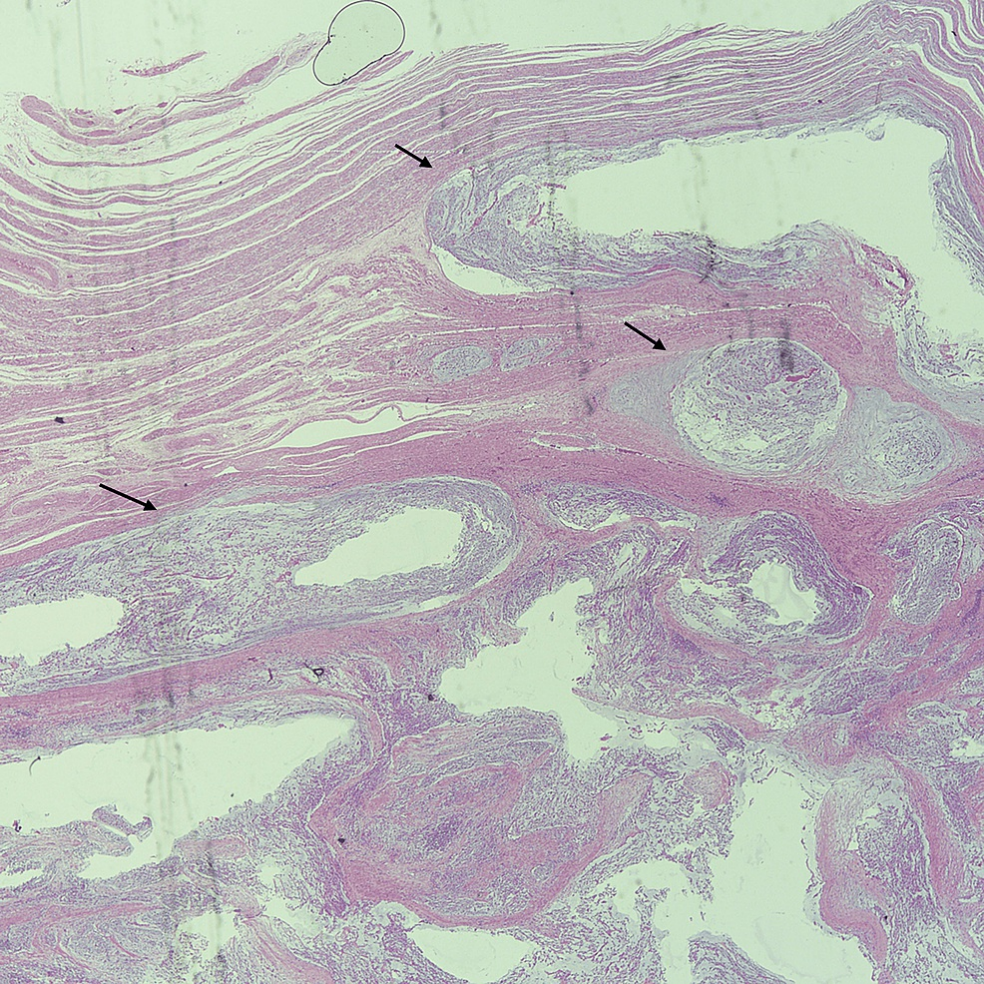
Hematoxylin and eosin stain of the tumor. Low-power view showing myxoid tumor infiltrating the myometrium.

**Figure 5:**
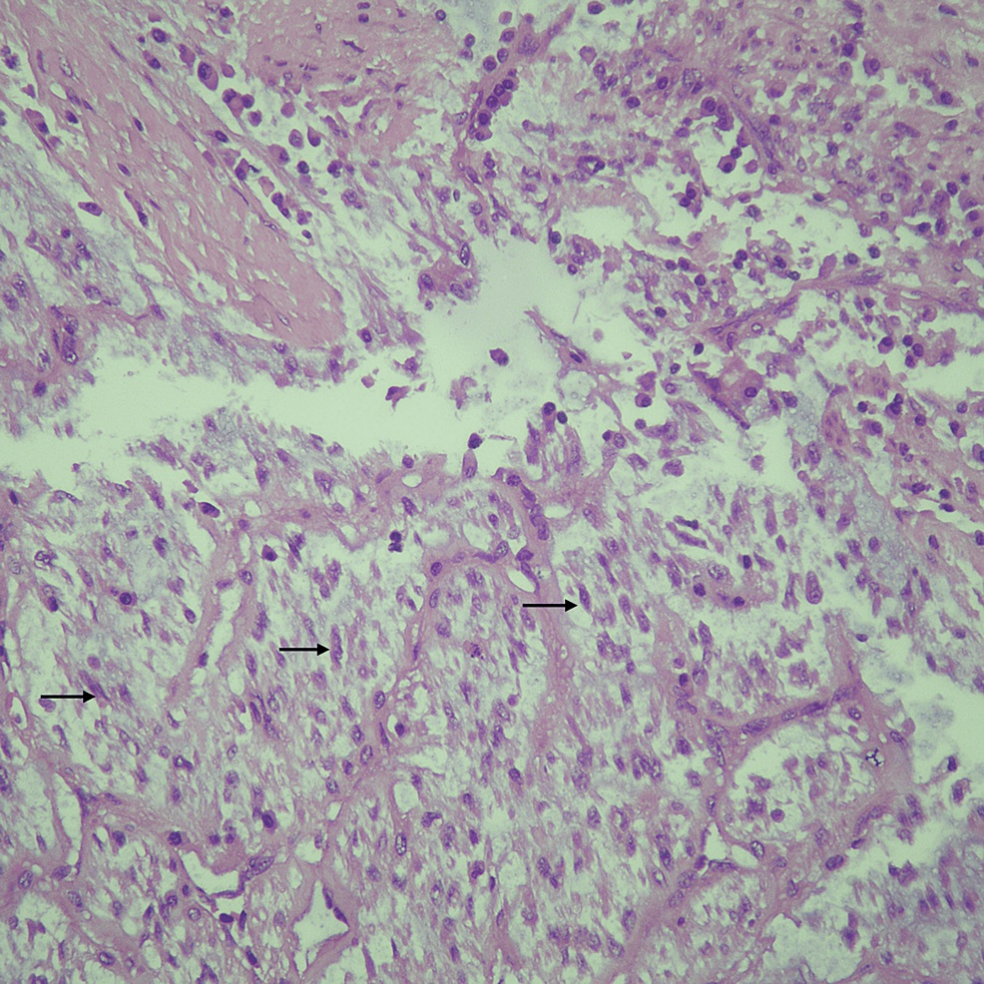
Hematoxylin and eosin stain of the myxoid tumor. 40x magnification of the bland spindle tumor cells (arrows) admixed with inflammatory cells in a myxoid background.

**Figure 6:**
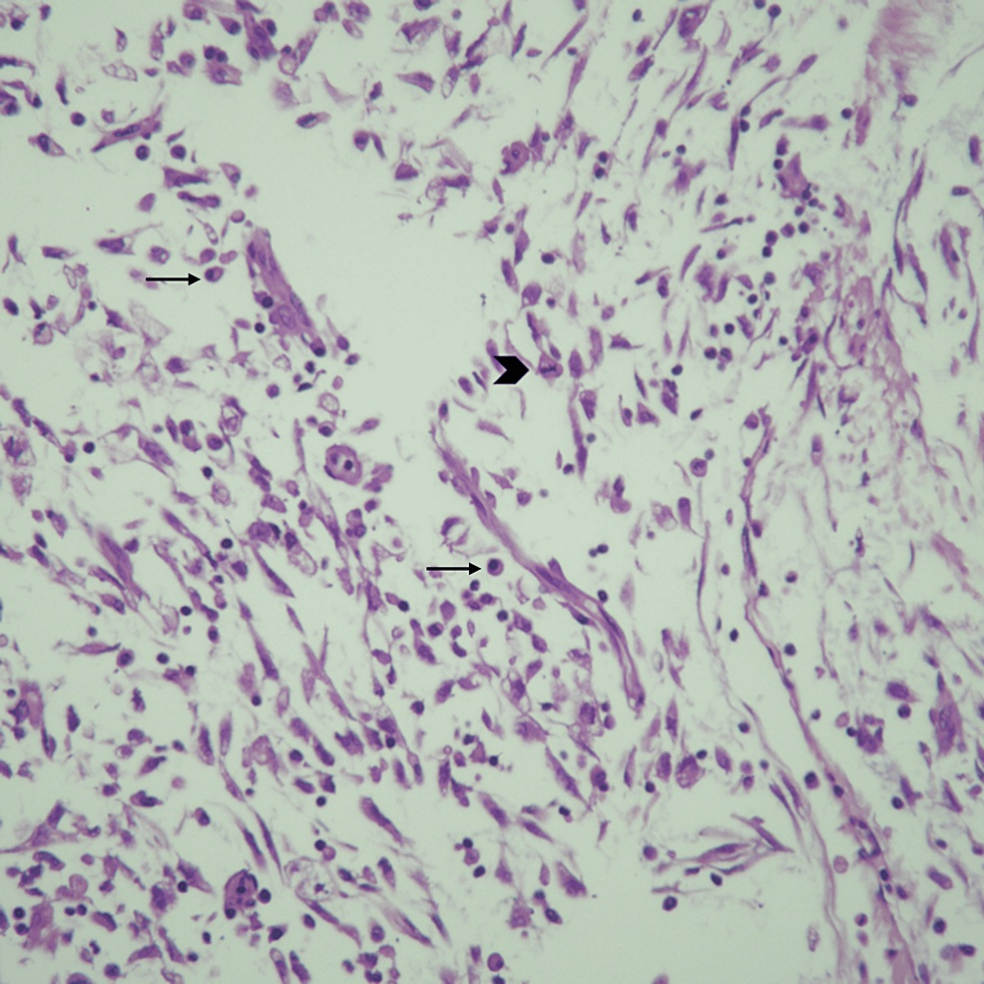
Hematoxylin and eosin stain of the uterine inflammatory myofibroblastic tumor (IMT). 40x magnification demonstrating plasma cells (arrows) and mitosis (arrowhead).

On microscopic examination, the luminal mass showed mildly atypical spindle cells and mild to moderate plasma cell predominant inflammatory infiltrate, with a mucinous and edematous background. The tumor infiltrated over 50% of the myometrium in a finger-like invasion pattern. Necrosis was present, and mitosis was minimal at around 2 per 10 high-power fields (Figures 4-6).

**Figure 7:**
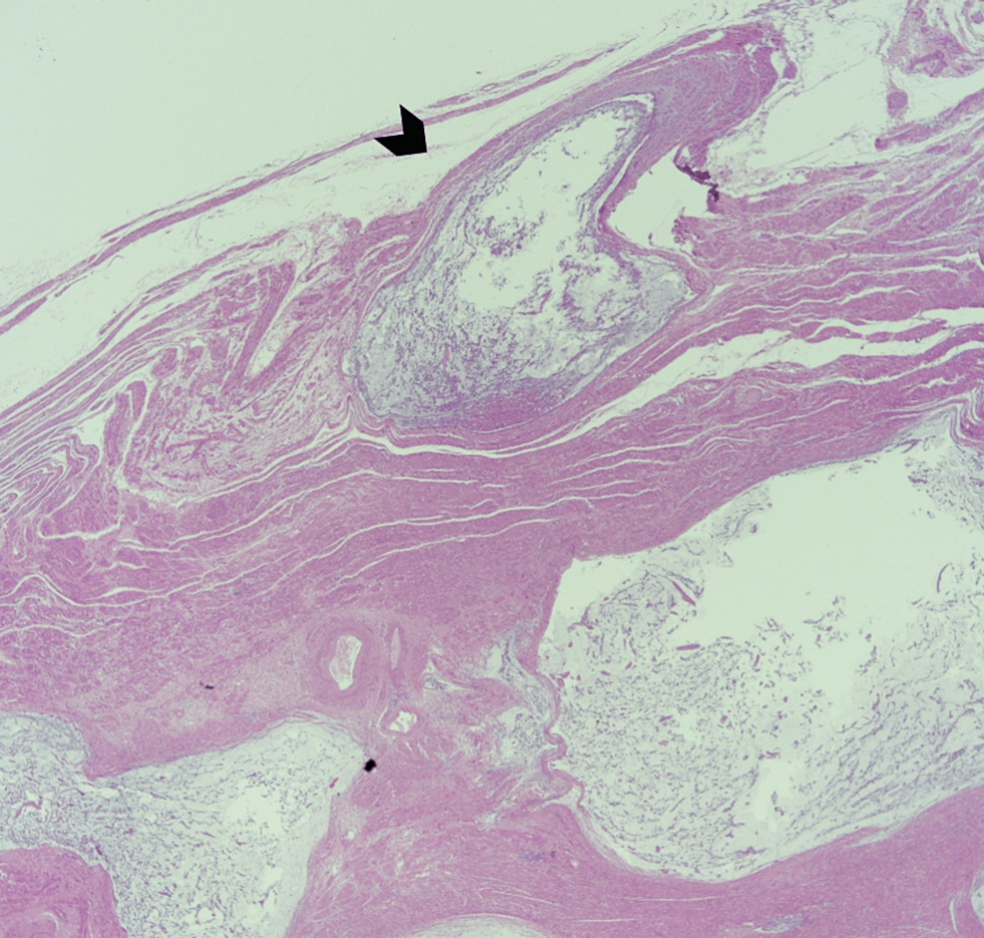
Vascular invasion of the inflammatory myofibroblastic tumor (IMT) is well-demonstrated in hematoxylin and eosin stain.

**Figure 8:**
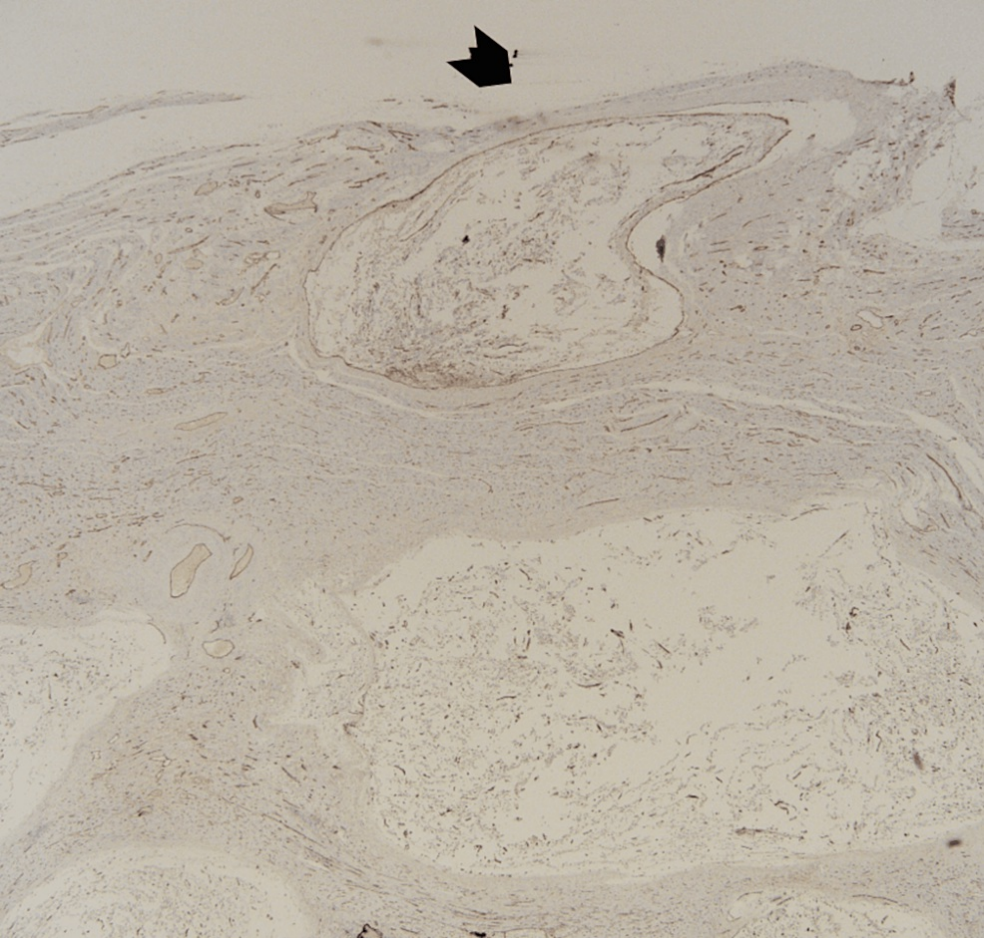
CD34 stain highlighting the vascular invasion.

**Figure 9:**
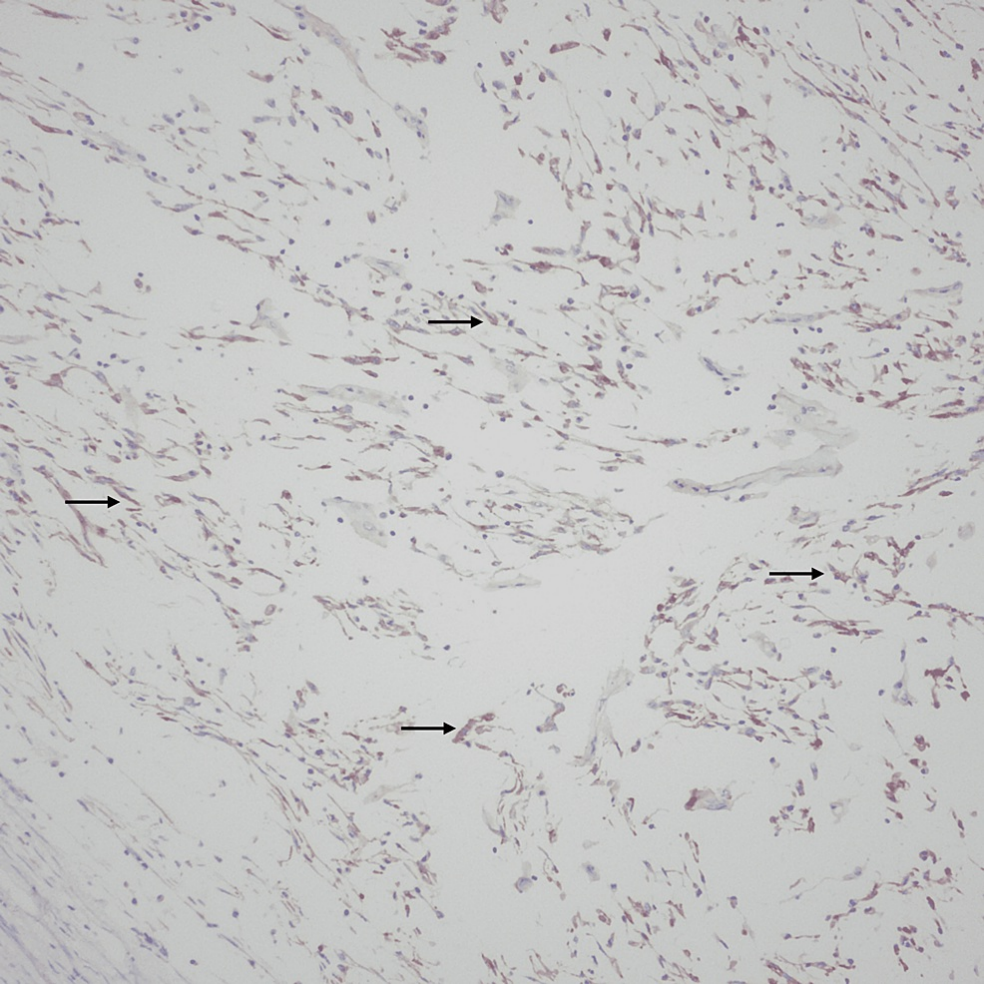
Immunohistochemical stain. The tumor cells are positive for anaplastic lymphoma kinase (ALK).

**Figure 10:**
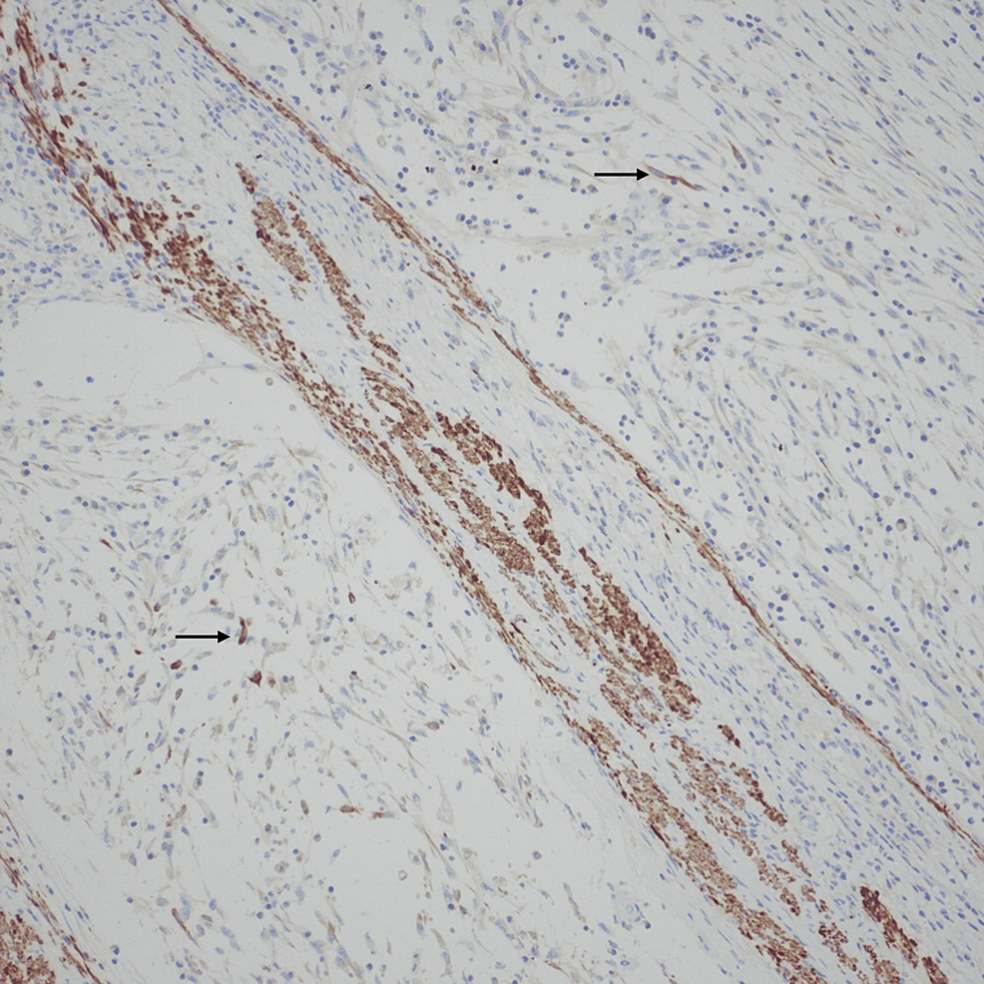
Immunohistochemical stain. The tumor cells are focally positive for desmin.

**Figure 11:**
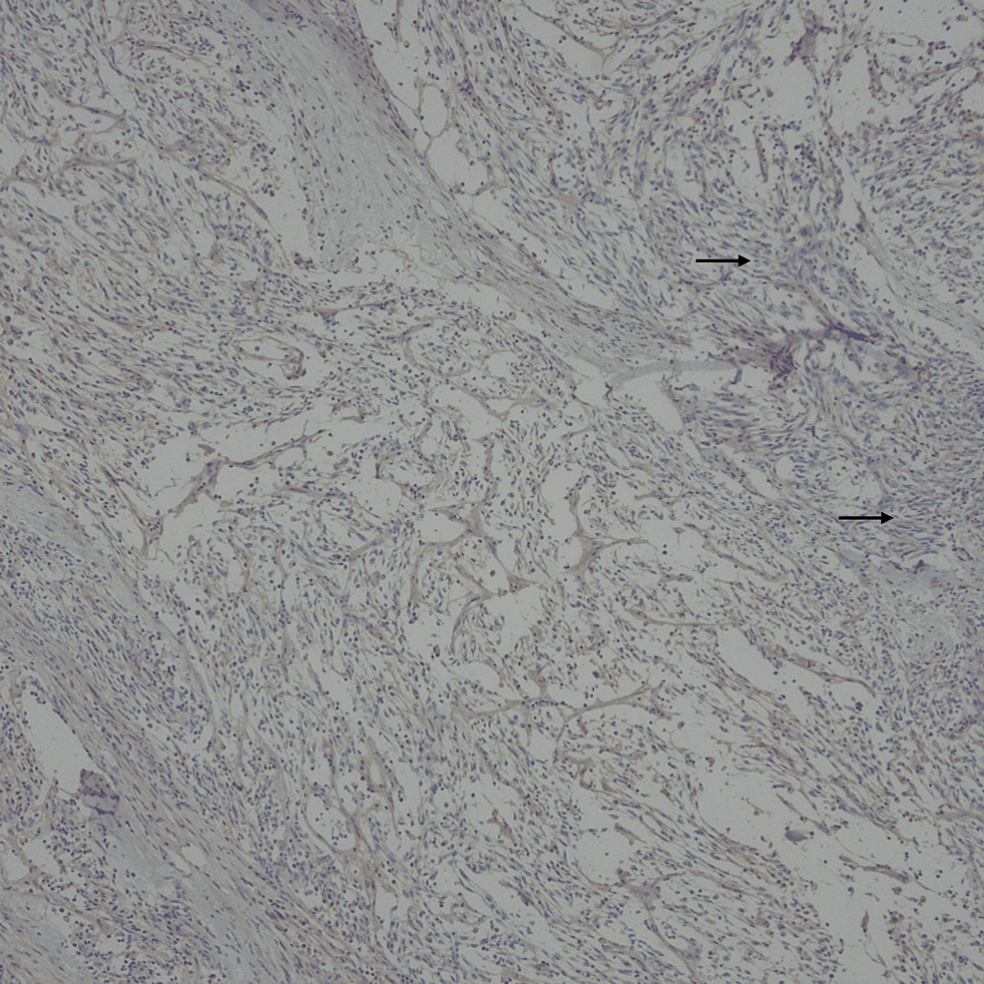
Immunohistochemical stain. Estrogen receptor (ER) is negative in the tumor cells.

Additionally, extensive vascular invasion was revealed by CD31 and CD34 immunohistochemical stains (Figures 7, 8). The tumor cells showed granular cytoplasmic positivity to anaplastic lymphoma kinase (ALK) and focal positivity to desmin and smooth muscle actin, whereas they were negative for caldesmon, estrogen receptor, P16, CD10, and cyclin D1 (Figures 9-11).

**Figure 12:**
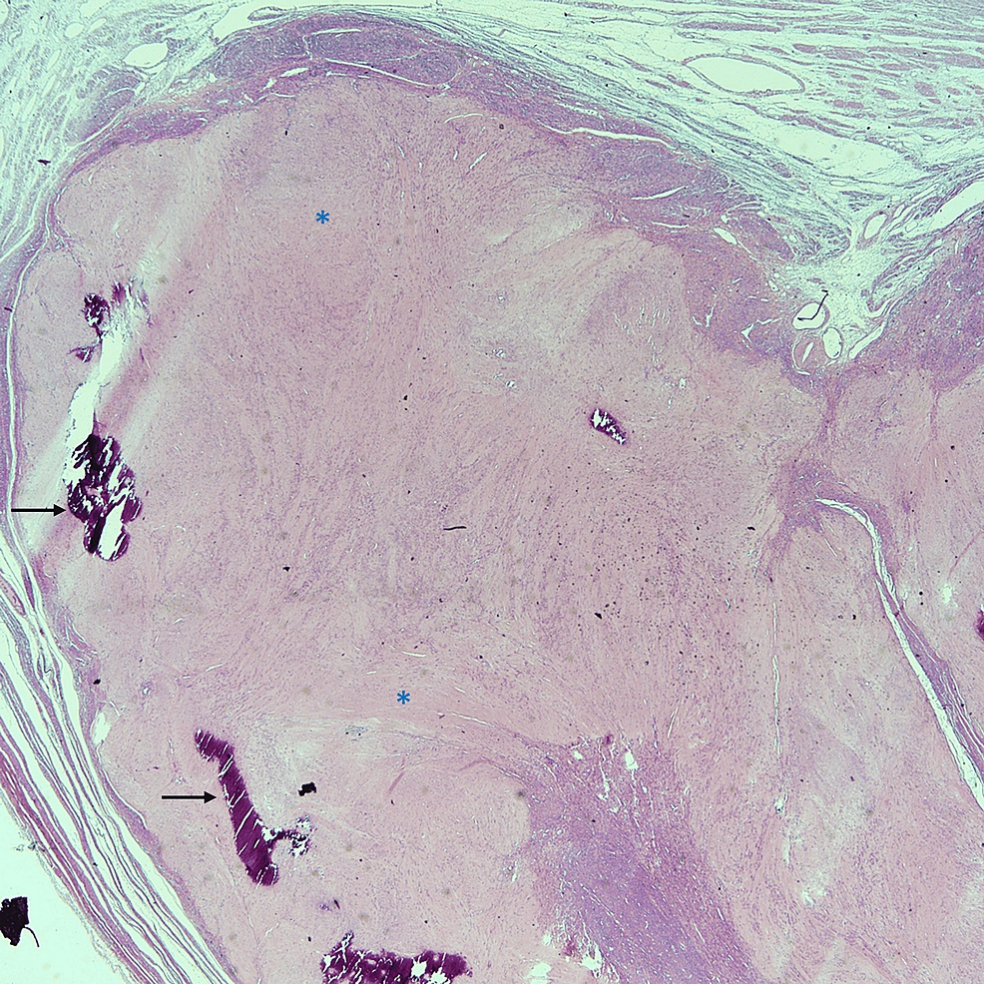
Hematoxylin and eosin stain of the subserosal leiomyoma with hyalinization (asterisks) and calcification (arrows).

Based on the microscopic features and immunohistochemistry findings, we diagnosed uterine IMT. The subserosal masses showed classic leiomyomata with hyalinization and dystrophic calcification (Figure 12).

The patient was transferred to an outside gynecology-oncology center. She later underwent a follow-up computer tomography (CT) scan with no significant findings and received no further treatment.

## Discussion

IMTs are soft-tissue neoplasms generally considered intermediate (rarely metastasizing) fibroblastic and myofibroblastic tumors. Although IMTs can occur in any organ, they are rarely seen in the female genital tract. To date, less than 100 cases of pure uterine IMTs have been reported in patients aged 3-78 years. Most IMTs manifest with abdominal pain, abdominal distension, and vaginal bleeding, and virtually all arise in the uterine corpus, with only seven cases reporting IMTs in the cervix [2-5]. ALK gene rearrangement was detected in around 80% of IMT cases, and no certain risk factors are linked to IMTs [3]. Radiological appearance is non-specific, and there is no gold standard imaging method for diagnosing uterine IMT [5].

We present a case of uterine IMT in a 60-year-old woman originally diagnosed with fibroids. Histologic analysis showed cells typical of an IMT (i.e., bland spindle cells mixed with inflammatory cells in a pool of mucin). The diagnosis was confirmed by immunohistochemistry. In most reported gynecologic IMT cases, the neoplastic myofibroblast cells show bland morphology with few mitoses, and necrosis is common. In a study by Bennet et al., the lymphovascular invasion was found in two of 13 cases of IMT [2], whereas no lymphovascular invasion was identified in nine cases described by Collins et al. [6]. Aggressive behaviors such as distal metastasis and death from the tumor are reported only in ALK-negative IMT cases. No other histologic features or diagnostic and predictive markers are correlated with adverse outcomes [7]. Our ALK-positive IMT case exhibited an infiltrative pattern and vascular invasion, which are considered unusual for this type of tumor. Given these concerning features, a six-month post-surgical CT scan was obtained and showed no sign of disease.

In cases of uterine tumors, the differential diagnosis must always include leiomyosarcoma, which is the most common malignant uterine mesenchymal neoplasm [8]. Among all leiomyosarcomas, the myxoid type is most similar to IMT in terms of bland morphology and low mitotic count. Myxoid leiomyoma is also considered, though it never shows an infiltrative pattern or lymphovascular invasion. In contrast, classic leiomyosarcoma has more prominent cytological atypia and pleomorphisms in addition to abundant mitosis [8,9]. Another valid differential diagnosis is myxoid endometrial stromal sarcoma. In this case, the combination of smooth muscle actin and ALK expression, along with the under-expression of desmin and absence of caldesmon, CD10, and ER staining, support the diagnosis of IMT. The presence of leiomyomata in the same uterus neither alters our differential diagnosis nor makes leiomyosarcoma more likely, as the literature reports only a few cases of leiomyosarcoma on leiomyomata, and none are myxoid [10]. To our knowledge, there are no reported cases of uterine IMT associated with leiomyomata, so we cannot confirm any relationship between leiomyomata and IMT. Detection of ALK rearrangement by fluorescence in situ hybridization can help confirm the diagnosis [6]. Because IMTs have no specific clinical or radiological features, they are often misdiagnosed as fibroids clinically.​​​​

## Conclusions

IMT is a rare uterine mesenchymal tumor with low malignant potential. Extensive sampling was crucial for a correct diagnosis in our case, as is the case with any potential uterine myxoid tumor. An immunohistochemistry panel is also essential to exclude differential diagnoses. This case highlights that not every benign-looking uterine tumor is a fibroid, and unexpected malignancies should always be considered when dealing with hysterectomy specimens.
